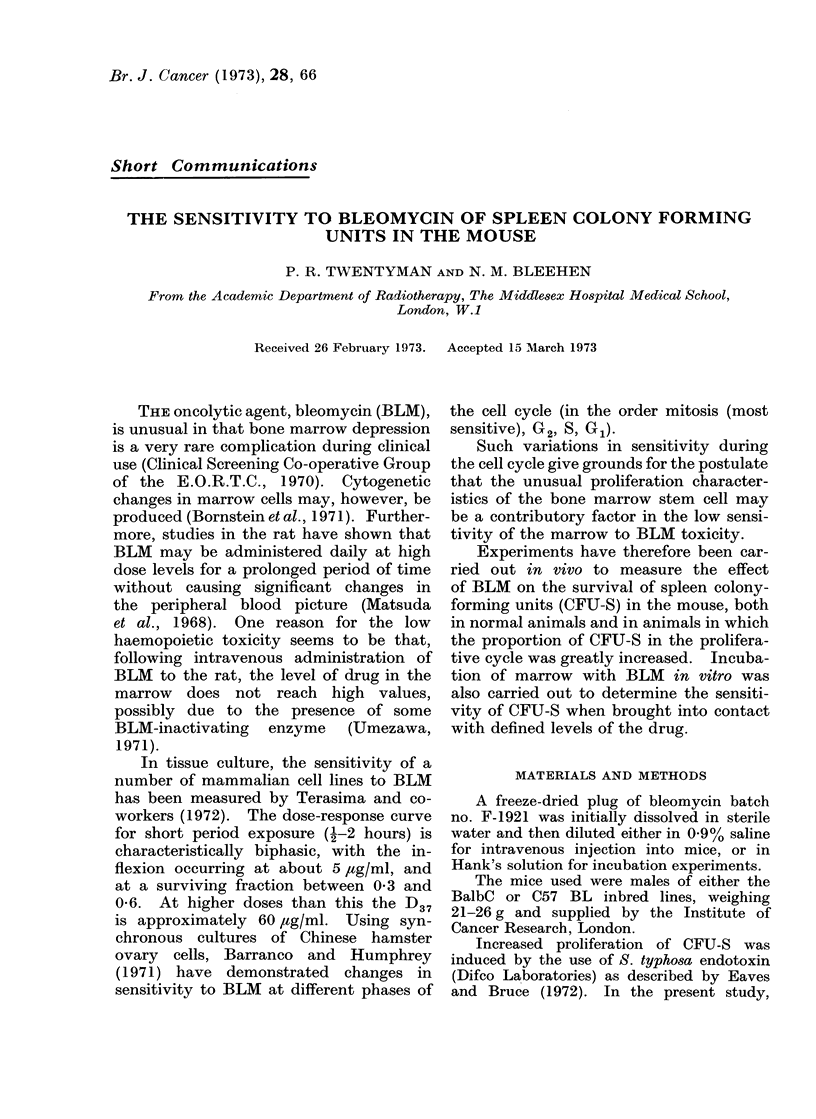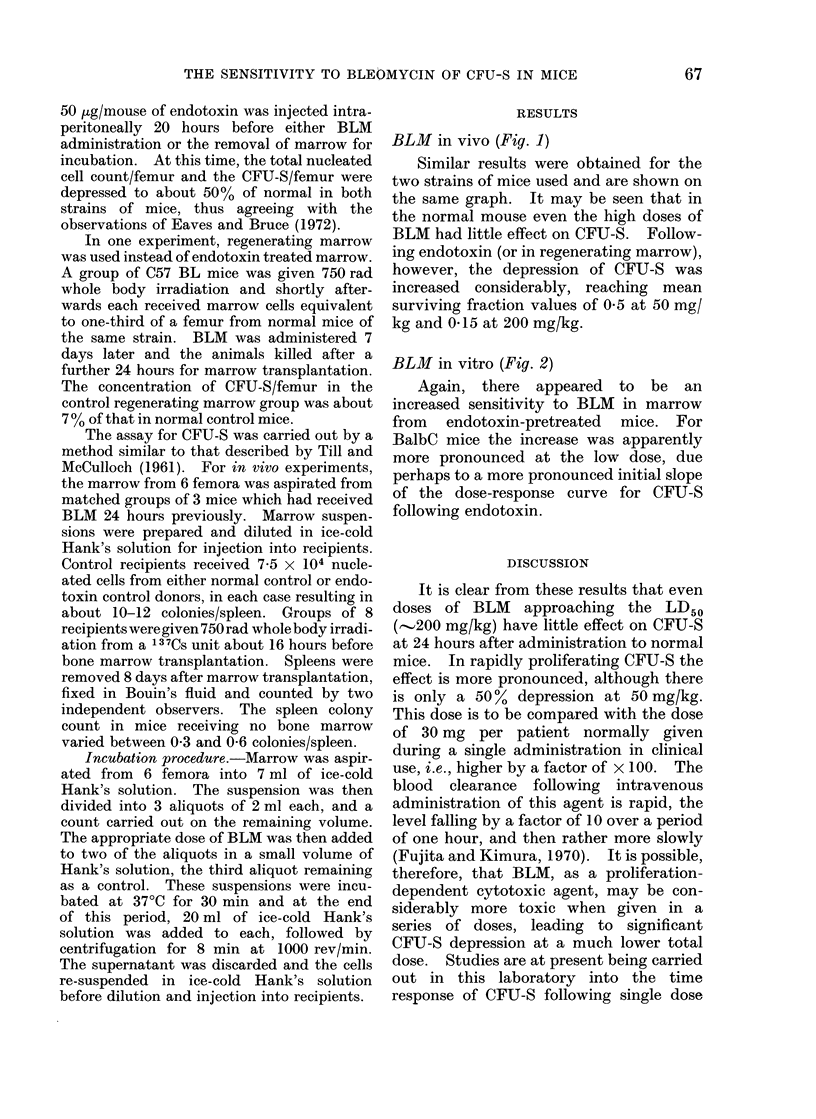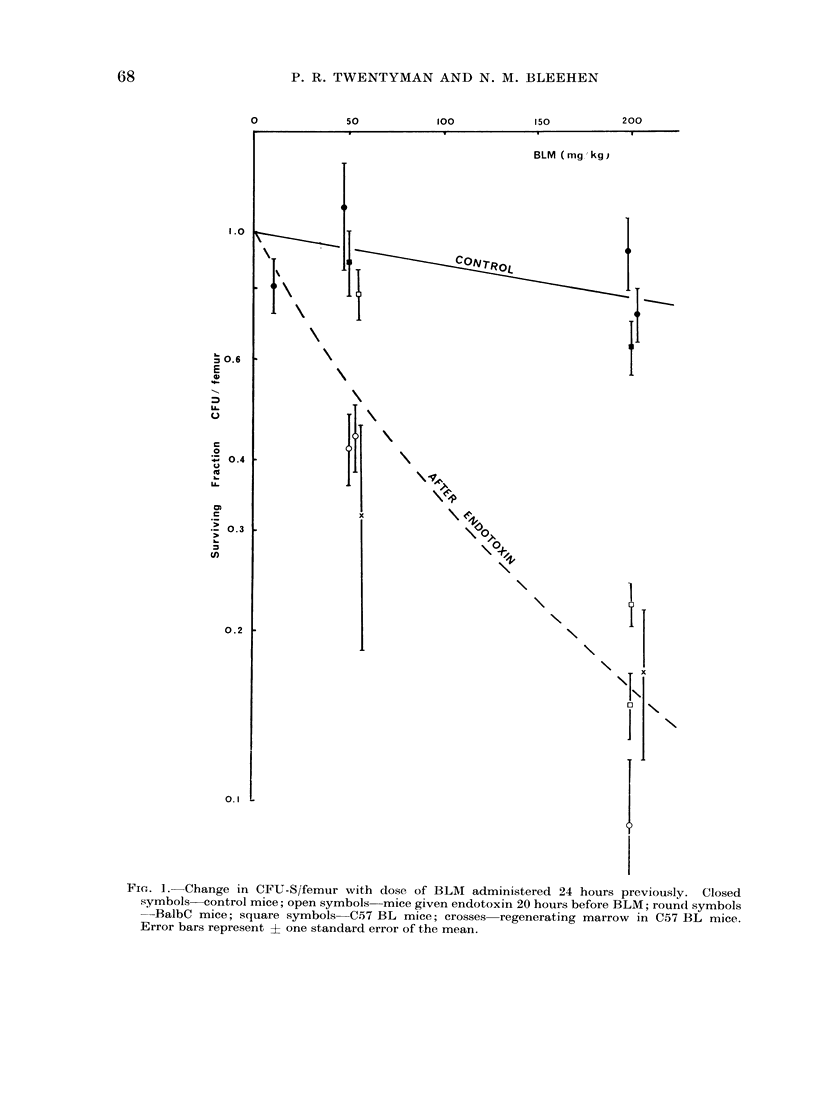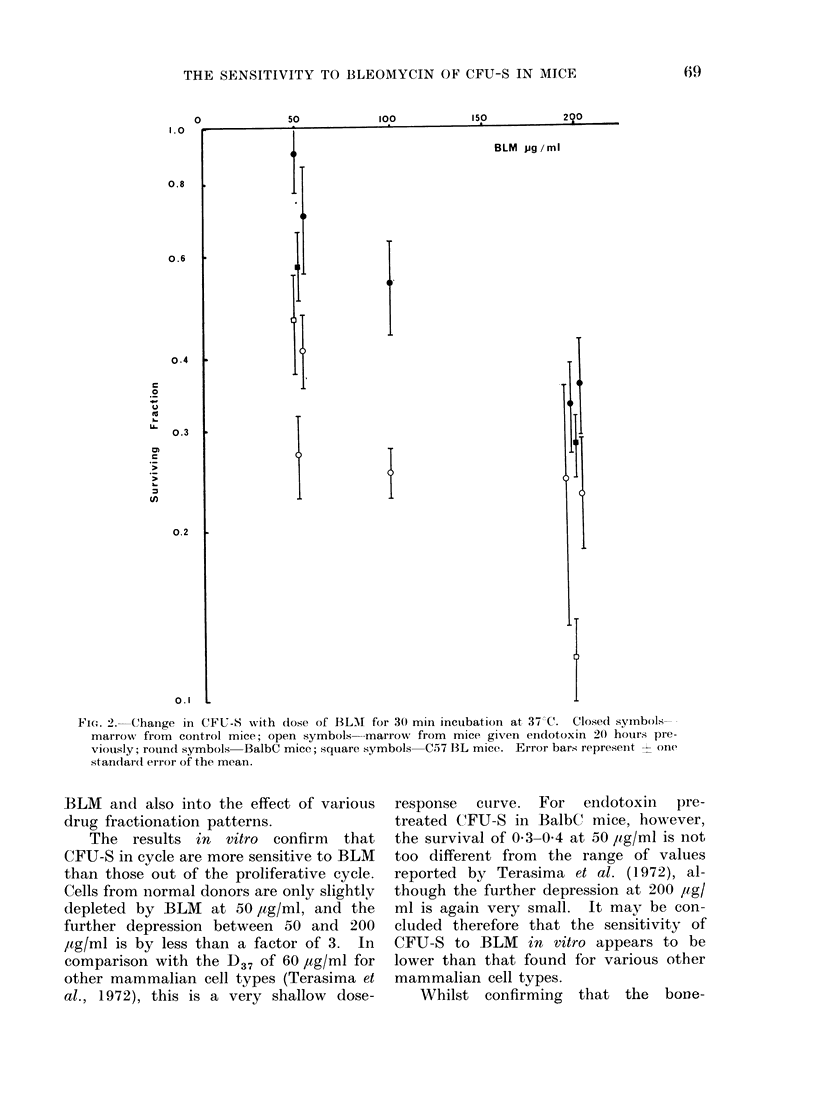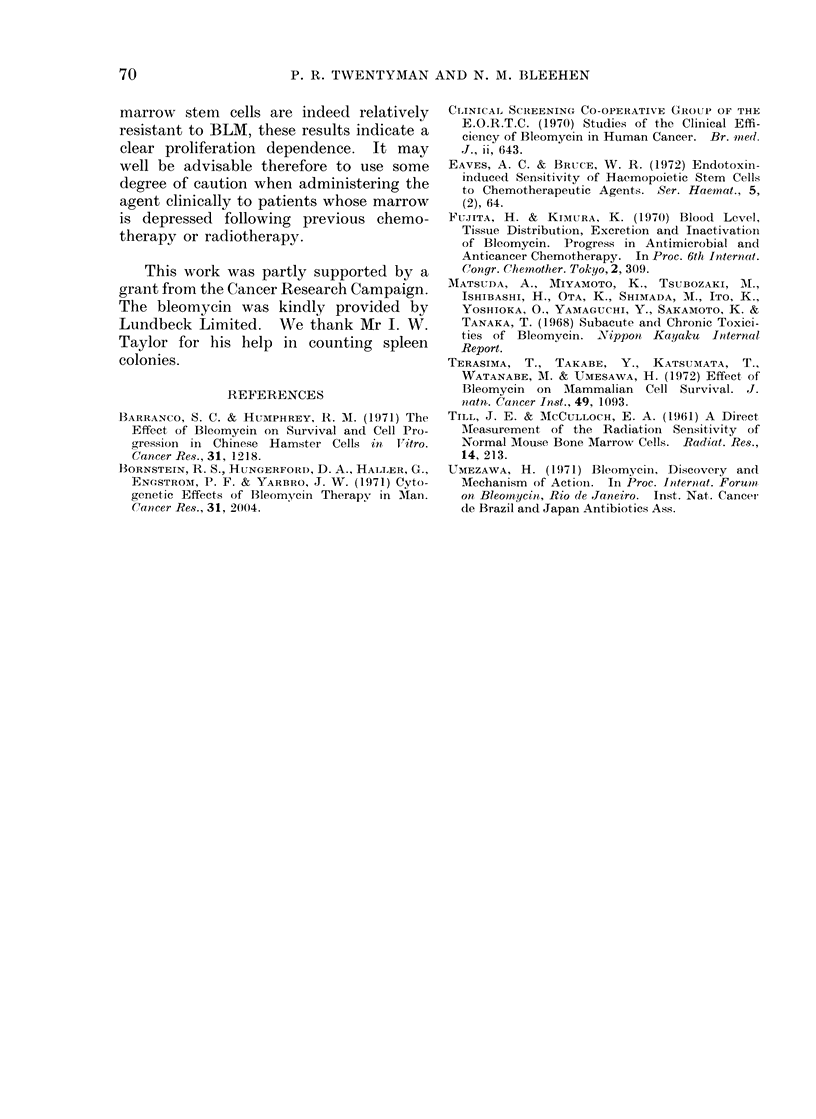# The sensitivity to bleomycin of spleen colony forming units in the mouse.

**DOI:** 10.1038/bjc.1973.71

**Published:** 1973-07

**Authors:** P. R. Twentyman, N. M. Bleehen


*Br. J. Cancer* (1973), **28**, 66

### *Short Communications*

### THE SENSITIVITY TO BLEOMYCIN OF SPLEEN COLONY FORMING UNITS IN THE MOUSE

P. R. TWENTYMAN AND N. M. BLEEHEN

*From the Academic Department of Radiotherapy, The Middlesex Hospital Medical School, London, W.1*

Received 26 February 1973. Accepted 15 March 1973

THE oncolytic agent, bleomycin (BLM), is unusual in that bone marrow depression is a very rare complication during clinical use (Clinical Screening Co-operative Group of the E.O.R.T.C., 1970). Cytogenetic changes in marrow cells may, however, be produced (Bornstein *et al.*, 1971). Furthermore, studies in the rat have shown that BLM may be administered daily at high dose levels for a prolonged period of time without causing significant changes in the peripheral blood picture (Matsuda *et al.*, 1968). One reason for the low haemopoietic toxicity seems to be that, following intravenous administration of BLM to the rat, the level of drug in the marrow does not reach high values, possibly due to the presence of some BLM-inactivating enzyme (Umezawa, 1971).

In tissue culture, the sensitivity of a number of mammalian cell lines to BLM has been measured by Terasima and coworkers (1972). The dose-response curve for short period exposure ($\frac{1}{2}$–2 hours) is characteristically biphasic, with the inflexion occurring at about 5 $\mu$g/ml, and at a surviving fraction between 0·3 and 0·6. At higher doses than this the $D_{37}$ is approximately 60 $\mu$g/ml. Using synchronous cultures of Chinese hamster ovary cells, Barranco and Humphrey (1971) have demonstrated changes in sensitivity to BLM at different phases of the cell cycle (in the order mitosis (most sensitive), $G_2$, S, $G_1$).

Such variations in sensitivity during the cell cycle give grounds for the postulate that the unusual proliferation characteristics of the bone marrow stem cell may be a contributory factor in the low sensitivity of the marrow to BLM toxicity.

Experiments have therefore been carried out *in vivo* to measure the effect of BLM on the survival of spleen colony-forming units (CFU-S) in the mouse, both in normal animals and in animals in which the proportion of CFU-S in the proliferative cycle was greatly increased. Incubation of marrow with BLM *in vitro* was also carried out to determine the sensitivity of CFU-S when brought into contact with defined levels of the drug.

### MATERIALS AND METHODS

A freeze-dried plug of bleomycin batch no. F-1921 was initially dissolved in sterile water and then diluted either in 0·9% saline for intravenous injection into mice, or in Hank's solution for incubation experiments.

The mice used were males of either the BalbC or C57 BL inbred lines, weighing 21–26 g and supplied by the Institute of Cancer Research, London.

Increased proliferation of CFU-S was induced by the use of *S. typhosa* endotoxin (Difco Laboratories) as described by Eaves and Bruce (1972). In the present study,

50 μg/mouse of endotoxin was injected intraperitoneally 20 hours before either BLM administration or the removal of marrow for incubation. At this time, the total nucleated cell count/femur and the CFU-S/femur were depressed to about 50% of normal in both strains of mice, thus agreeing with the observations of Eaves and Bruce (1972).

In one experiment, regenerating marrow was used instead of endotoxin treated marrow. A group of C57 BL mice was given 750 rad whole body irradiation and shortly afterwards each received marrow cells equivalent to one-third of a femur from normal mice of the same strain. BLM was administered 7 days later and the animals killed after a further 24 hours for marrow transplantation. The concentration of CFU-S/femur in the control regenerating marrow group was about 7% of that in normal control mice.

The assay for CFU-S was carried out by a method similar to that described by Till and McCulloch (1961). For *in vivo* experiments, the marrow from 6 femora was aspirated from matched groups of 3 mice which had received BLM 24 hours previously. Marrow suspensions were prepared and diluted in ice-cold Hank's solution for injection into recipients. Control recipients received $7{\cdot}5 \times 10^4$ nucleated cells from either normal control or endotoxin control donors, in each case resulting in about 10–12 colonies/spleen. Groups of 8 recipients were given 750 rad whole body irradiation from a $^{137}$Cs unit about 16 hours before bone marrow transplantation. Spleens were removed 8 days after marrow transplantation, fixed in Bouin's fluid and counted by two independent observers. The spleen colony count in mice receiving no bone marrow varied between 0·3 and 0·6 colonies/spleen.

*Incubation procedure.*—Marrow was aspirated from 6 femora into 7 ml of ice-cold Hank's solution. The suspension was then divided into 3 aliquots of 2 ml each, and a count carried out on the remaining volume. The appropriate dose of BLM was then added to two of the aliquots in a small volume of Hank's solution, the third aliquot remaining as a control. These suspensions were incubated at 37°C for 30 min and at the end of this period, 20 ml of ice-cold Hank's solution was added to each, followed by centrifugation for 8 min at 1000 rev/min. The supernatant was discarded and the cells re-suspended in ice-cold Hank's solution before dilution and injection into recipients.

### RESULTS

### *BLM* in vivo (*Fig. 1*)

Similar results were obtained for the two strains of mice used and are shown on the same graph. It may be seen that in the normal mouse even the high doses of BLM had little effect on CFU-S. Following endotoxin (or in regenerating marrow), however, the depression of CFU-S was increased considerably, reaching mean surviving fraction values of 0·5 at 50 mg/kg and 0·15 at 200 mg/kg.

### *BLM* in vitro (*Fig. 2*)

Again, there appeared to be an increased sensitivity to BLM in marrow from endotoxin-pretreated mice. For BalbC mice the increase was apparently more pronounced at the low dose, due perhaps to a more pronounced initial slope of the dose-response curve for CFU-S following endotoxin.

### DISCUSSION

It is clear from these results that even doses of BLM approaching the $LD_{50}$ (~200 mg/kg) have little effect on CFU-S at 24 hours after administration to normal mice. In rapidly proliferating CFU-S the effect is more pronounced, although there is only a 50% depression at 50 mg/kg. This dose is to be compared with the dose of 30 mg per patient normally given during a single administration in clinical use, *i.e.*, higher by a factor of × 100. The blood clearance following intravenous administration of this agent is rapid, the level falling by a factor of 10 over a period of one hour, and then rather more slowly (Fujita and Kimura, 1970). It is possible, therefore, that BLM, as a proliferation-dependent cytotoxic agent, may be considerably more toxic when given in a series of doses, leading to significant CFU-S depression at a much lower total dose. Studies are at present being carried out in this laboratory into the time response of CFU-S following single dose

FIG. 1.—Change in CFU-S/femur with dose of BLM administered 24 hours previously. Closed symbols—control mice; open symbols—mice given endotoxin 20 hours before BLM; round symbols—BalbC mice; square symbols—C57 BL mice; crosses—regenerating marrow in C57 BL mice. Error bars represent $\pm$ one standard error of the mean.

FIG. 2.—Change in CFU-S with dose of BLM for 30 min incubation at 37°C. Closed symbols—marrow from control mice; open symbols—marrow from mice given endotoxin 20 hours previously; round symbols—BalbC mice; square symbols—C57 BL mice. Error bars represent ± one standard error of the mean.

BLM and also into the effect of various drug fractionation patterns.

The results *in vitro* confirm that CFU-S in cycle are more sensitive to BLM than those out of the proliferative cycle. Cells from normal donors are only slightly depleted by BLM at 50 μg/ml, and the further depression between 50 and 200 μg/ml is by less than a factor of 3. In comparison with the $D_{37}$ of 60 μg/ml for other mammalian cell types (Terasima *et al.*, 1972), this is a very shallow dose-response curve. For endotoxin pretreated CFU-S in BalbC mice, however, the survival of 0·3–0·4 at 50 μg/ml is not too different from the range of values reported by Terasima *et al.* (1972), although the further depression at 200 μg/ml is again very small. It may be concluded therefore that the sensitivity of CFU-S to BLM *in vitro* appears to be lower than that found for various other mammalian cell types.

Whilst confirming that the bonemarrow stem cells are indeed relatively resistant to BLM, these results indicate a clear proliferation dependence. It may well be advisable therefore to use some degree of caution when administering the agent clinically to patients whose marrow is depressed following previous chemotherapy or radiotherapy.

This work was partly supported by a grant from the Cancer Research Campaign. The bleomycin was kindly provided by Lundbeck Limited. We thank Mr I. W. Taylor for his help in counting spleen colonies.

### REFERENCES

BARRANCO, S. C. & HUMPHREY, R. M. (1971) The Effect of Bleomycin on Survival and Cell Progression in Chinese Hamster Cells *in Vitro*. *Cancer Res.*, **31**, 1218.

BORNSTEIN, R. S., HUNGERFORD, D. A., HALLER, G., ENGSTROM, P. F. & YARBRO, J. W. (1971) Cytogenetic Effects of Bleomycin Therapy in Man. *Cancer Res.*, **31**, 2004.

CLINICAL SCREENING CO-OPERATIVE GROUP OF THE E.O.R.T.C. (1970) Studies of the Clinical Efficiency of Bleomycin in Human Cancer. *Br. med. J.*, ii, 643.

EAVES, A. C. & BRUCE, W. R. (1972) Endotoxin-induced Sensitivity of Haemopoietic Stem Cells to Chemotherapeutic Agents. *Ser. Haemat.*, **5**, (2), 64.

FUJITA, H. & KIMURA, K. (1970) Blood Level, Tissue Distribution, Excretion and Inactivation of Bleomycin. Progress in Antimicrobial and Anticancer Chemotherapy. In *Proc. 6th Internat. Congr. Chemother. Tokyo*, **2**, 309.

MATSUDA, A., MIYAMOTO, K., TSUBOZAKI, M., ISHIBASHI, H., OTA, K., SHIMADA, M., ITO, K., YOSHIOKA, O., YAMAGUCHI, Y., SAKAMOTO, K. & TANAKA, T. (1968) Subacute and Chronic Toxicities of Bleomycin. *Nippon Kayaku Internal Report.*

TERASIMA, T., TAKABE, Y., KATSUMATA, T., WATANABE, M. & UMESAWA, H. (1972) Effect of Bleomycin on Mammalian Cell Survival. *J. natn. Cancer Inst.*, **49**, 1093.

TILL, J. E. & MCCULLOCH, E. A. (1961) A Direct Measurement of the Radiation Sensitivity of Normal Mouse Bone Marrow Cells. *Radiat. Res.*, **14**, 213.

UMEZAWA, H. (1971) Bleomycin, Discovery and Mechanism of Action. In *Proc. Internat. Forum on Bleomycin, Rio de Janeiro.* Inst. Nat. Cancer de Brazil and Japan Antibiotics Ass.